# Ocular findings from otoneurological examinations in children with and without dyslexia: a systematic review with meta-analysis

**DOI:** 10.1016/j.bjorl.2021.10.006

**Published:** 2021-11-25

**Authors:** Ysa Karen dos Santos Macambira, Jessyca Vanessa dos Santos Barbosa, Bianca Manchester de Queiroga, Ana Augusta de Andrade Cordeiro, Denise Costa Menezes, Maria Luiza Lopes Timóteo de Lima, Karina Paes Advíncula

**Affiliations:** a Universidade Federal de Pernambuco, Departamento de Fonoaudiologia, Recife, PE, Brazil; b Universidade Federal de Pernambuco (UFPE), Programa de Pós-Graduação em Comunicação Humana em Saúde, Recife, PE, Brazil; c Universidade Federal de Pernambuco (UFPE), Curso de Fonoaudiologia, Recife, PE, Brazil

**Keywords:** Dyslexia, Disorder of language, Video-nystagmography, Vectoelectro-nystagmography, Eye tests

## Abstract

**Objective:**

To investigate ocular movements measures of vectoelectro-nystagmography and video-nystagmography in dyslexic children and compare with measures of typical children.

**Methods:**

A systematic review of observational studies comparing the ocular movements differences between dyslexic and typical children with no publication date or language restriction. The literature survey included the bibliographic databases MEDLINE, ScienceDirect, Scopus, Web of Science, SciELO, Lilacs and CENTRAL. Gray literature databases were also searched, including: OpenGrey.eu, DissOnline.de, The New York Academy of Medicine and WorldCat. The meta-analysis was performed using software RevMan 5.3 (Cochran Collaboration).

**Results:**

A total of 2375 articles were found of which 113 fell within the inclusion criteria. Among these, 52 were duplicates (found in more than one research source), and 45 articles were selected for reading in full. Thirteen (13) articles were included for analysis and discussion. Meta-analysis showed statistical differences between the two groups for the total number of saccades and duration of fixation.

**Conclusion:**

The study revealed that children with dyslexia have longer duration of fixation and fewer saccades during ocular movements on vectoelectro-nystagmography and video-nystagmography when compared to children without dyslexia.

## Highlights

• Vestibular evaluation of children with dyslexia.
• Ocular evidence of otoneurology in dyslexia.
• The measurements of ocular tests in children with dyslexia.
• Vectoelectro-nystagmography and videonystagmography exams in children with dyslexia.

## Introduction

Dyslexia is considered a specific neurobiological learning disorder that affects basic reading and language skills. It occurs due to differences in the functioning of brain systems responsible for phonological processing that result in difficulty in processing the sounds of words and associating them with the letters or sequences of letters that represent them. Other factors that may be associated are deficits in executive functions, difficulties in auditory and/or visual processing and psychomotor development.

It is considered a specific learning disorder because its symptoms usually affect the academic performance of students without any other alteration (neurological, sensory or motor) to justify them.^1^

Reading and writing processes involve complex and independents functions, in which cognitive and motor abilities are used. These functions result in a correct writing and a correct word decoding during reading.^2^, ^3^ Another important ability for reading and writing is the visual-motor perception, that coordenates visual information and motor programming.^4^

The importance of eyes movement for the reading and writing learing processes has been studied.^5^, ^6^, ^7^, ^8^ It is not only visual accurance that it matters, but also the visual-motor behavior characterized by sacadde alternated eyes movements, tracing and fixation.^9^

The eyes movements acuity directly interferes on the visual fixation of the word during reading and may be altered in people with dyslexia. Different bahavior patterns of visual movements has been found in dyslexic individuals as they read.^10^, ^11^, ^12^

For an accurate vision, it is necessary to stabilize the image on the retina, even during head movement. In this case, there is a compensatory movement of the eyes to the opposite direction of the cephalic movement. This compensatory movement is called the Vestibulo-Ocular Reflex (VOR) and involves the integration of the vestibular system and the extraocular muscles.^13^

This activity can be measured by objective measures such as Video-Nystagmography (VNG) and Vectoelectro-Nystagmography (VENG), which are used for the functional evaluation of the vestibular system.^7^, ^14^ Vectoelectro-nystagmography is one of the most used methods to record eye movements with greater diagnostic sensitivity. It allows the measurement of vestibulo-oculomotor function parameters, by comparing stimuli and responses. It also allows the identification of the ocular direction by recording the vestibulocochlear reflex, saccades, tracing, optokinetic and ocular fixation.^15^, ^16^, ^17^ Videonystagmography is a computerized system that uses infrared sensors with special glasses to record, measures and analyzes eye movements.^18^

The aim of this study was to investigate the measurements of ocular tests from vectoelectro-nystagmography and videonystagmography exams in children with dyslexia and to compare them with typical children.

## Methods

The aim of this study was to investigate ocular movements measures of vectoelectro-nystagmography and video-nystagmography in dyslexic children and compare with measures of typical children. In order to achieve it, a systematic review of the literature on observational studies was carried out in order to answer the following guiding question: “Do dyslexic children have different results in ocular tests of VNG and VENG when compared to typical children?”. The review was structured according to the items defined by the Preferred Reporting Items for Systematic Reviews and Meta-Analyzes Statement (Prisma)^19^ and a full protocol was published in the Prospero database (http://www.crd.york.ac.uk/PROSPERO) under the registration number CRD42018081954.

### Search strategy

The strategy started finding descriptors (DECs and MeSH) and Free Terms (TL) based on the first two elements of the PICo (Population, Interest, Context) present in the title. The search terms used were: (dyslexia OR developmental reading disorder OR word blindness OR developmental dyslexia OR reading disorder OR alexia OR language disorder) AND (otoneurology OR video nystagmography OR vectoelectronystagmography OR ocular tests OR ocular evidence OR nystagmus OR eye movement OR nystagmus semi spontaneous OR optokinetic nystagmus OR pendular tracking OR saccades). The complete strategy is found in the supplementary material (Supplementary Table 1).

The searches were conducted between January and July 2018 and revised in August of the same year. The following electronic bibliographic databases were searched: MEDLINE, Cochrane Central Register of Controlled Trials (CENTRAL), Latin American and Caribbean Literature in Health Sciences (LILACS), Scientific Electronic Library Online (SciELO), ScienceDirect, Scopus, Web of Science. The search of the gray literature was conducted at: OpenGrey.eu, DissOnline.de, The New York Academy of Medicine and WorldCat. There were no manual searches made for the included articles and experts in the area were not contacted to avoid the risk of citation bias.^20^

### Eligibility criteria

The inclusion criteria were children with dyslexia and with results of ocular tests that support VNG and VENG. The exclusion criteria were children with hearing loss, visual impairment, neurological alterations, other learning disorders, cognitive alterations and terms such as neglect dyslexia, pure alexia or hemianopia.

Repeated articles in different databases were also excluded. As a control group we considered: children with normal hearing, without any neurological problem, deficits in reading or writing, central auditory processing problems, as well as visual or cognitive problems. All submitted to ocular tests of VNG and VENG under similar conditions of the test group procedure. Finally, studies with at least a title and/or abstract in English were included, but there was no other restriction on language or date of publication.

### Extraction of data

This review was performed by two researchers who independently identified titles and abstracts extracted from electronic database sources that met the inclusion criteria. A third researcher would decide whenever there was a divergence between the two researchers, however, this was not needed as there was no divergence. Full texts of these potentially selected studies were entirety analyzed. The searched studies outcomes were differences in the mean values of saccadic amplitudes, duration of fixation and total number of saccades recorded by VNG and/or VENG in both group of children, associated to the specific complaint of the language disorder.

Data were analyzed from published articles and authors were contacted for additional information. In addition to the outcome data, the authors' names, title, year of publication, country, age groups of the groups and the number of subjects in each group were also extracted. A standard form for data storage was created based on the model adopted by Cochrane.^21^

### Bias risk assessment method

The risk of bias was assessed according to the recommendations of the handbook and the Newcastle-Ottawa scale, adapted for cross-sectional observational studies. The quality of the work was also evaluated independently by two researchers and the divergences were evaluated by consensus. The list of evaluated items used for scoring was: (1) Representativeness of the sample; (2) Sample size; (3) Management of non-responses; (4) Exposure calculation (risk factor); (5) Comparability, to investigate whether individuals in different groups of outcomes are comparable, based on study design or analysis, control of confounding factors; (6) Evaluation of results and (7) Statistical testing (Supplementary Table 2). The maximum score possible was 10 points.

### Data analysis method

The amplitude of the saccades, the duration of fixation and the total number of saccades measured in the ocular movements of the two groups were compared by meta-analysis. We used the effect of the mean difference between the groups as a measure and a model of random effects as a statistical method of analysis. A value of α less than 0.05 was considered statistically significant. When it was not possible to obtain adequate data for analysis, Cochrane’s recommendations were followed. Statistical heterogeneity between the studies was assessed using Cochrane's Q test and the presence of inconsistency was assessed with the I2 test A *p*-value < 0.10 was considered statistically significant. When necessary, study characteristics, considered to be potential sources of heterogeneity, were included in a subgroup analysis. In addition, in the case of heterogeneity, the studies were removed one by one to investigate whether a study was the source of heterogeneity. All analyses were conducted with RevMan 5.3 software (Cochrane Collaboration).^21^

## Results

### Included studies

**Figure 1:**
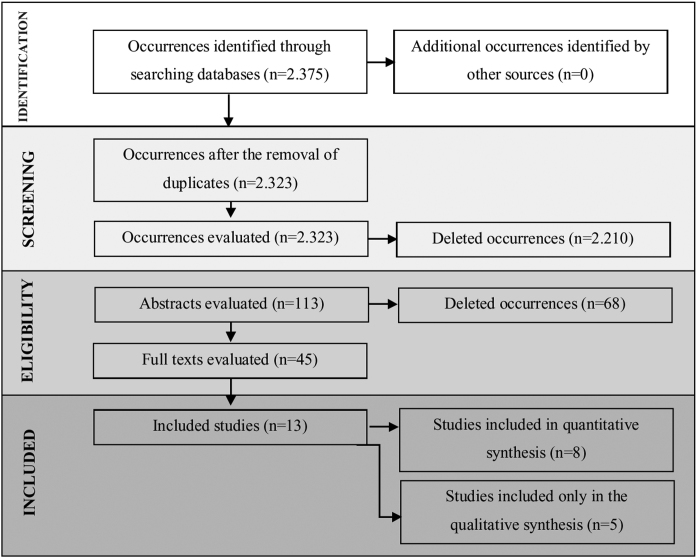
Flowchart showing processes used to search for and select articles.

**Figure 2:**
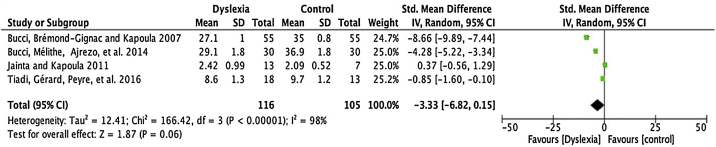
Meta-analysis: comparison of means and standard deviations of the amplitudes of saccades, for dyslexic children and for non-dyslexic children, per study.

**Figure 3:**
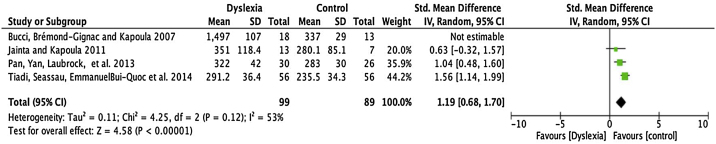
Meta-analysis: comparison of means and standard deviations of the durations of saccadi fixations, for dyslexic children and for non-dyslexic children, per study.

**Figure 4:**
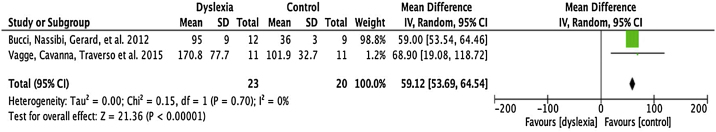
Meta-analysis: comparison of means and standard deviations of the total number of saccades, for dyslexic children and for non-dyslexic children, per study.

A flow diagram that illustrates the search and selection process is presented in Fig. 1. Of the 2375 titles considered relevant, 113 abstracts were selected to be read, and of these, 45 complete texts were selected for reading in full. After reading, 32 articles were excluded because they did not meet the eligibility criteria, five because they did not have enough data (Table 1). Therefore, 13 complete texts were included in the qualitative analysis and, of these, only 8 for quantitative meta-analysis (Table 2). The mean values of the saccade amplitudes, the duration of fixation and total number of saccades in children with and without dyslexia extracted from the selected articles are shown in Fig. 2 (Table 3), Fig. 3 (Table 4) and Fig. 4 (Table 5) respectively.

**Table 1:** Full texts excluded from the analysis.

Name	Location	Year	Reason	Name	Location	Year	Reason
Benfatto et al.	Sweden	2016	Did not analyze saccades	Bucci et al.^27^,^a^	France	2008	Insufficient data for meta-analysis
Krieber et al.	Austria	2016	Age group	Huang et al.	China	2008	Reviewed abstract only
Lukasova et al.^24^,^a^	Brazil	2016	Insufficient data for meta-analysis	Kirkby et al.	UK	2008	Review article
Huettig e Brouwer	UK	2015	Age group	Judge et al.	England	2007	Age group
Kunert e Sheepers	Scotland	2015	Did not analyze saccades	Wright	Australia	2007	Visual training
Kim	China	2014	Reviewed abstract only	Judge et al.	England	2006	Age group
Pan	China	2014	Did not analyze saccades	Santos et al.^13^,^a^	Brazil	1995	Insufficient data for meta-analysis
Bucci et al.^25^,^a^	France	2014	Insufficient data for meta-analysis	Rodger	Australia	1985	Review article
Xiu et al.	China	2013	Unsupported method	Brown et al.	USA	1983	Visual Evaluation
Pensiero et al.	Italy	2013	Age group	Olson et al.	Germany	1983	Unsupported method
Bellocchi e al.	France	2013	Review article	Romero et al.	USA	1982	Unsupported method
Oliveira et al.	Brazil	2013	Age group	Pavlidis	USA	1981	Visual Training
Kraljevic e Palmović^26^,^a^	Croatia	2011	Insufficient data for meta-analysis	Gabersek et al.	France	1981	Unsupported method
Quercia	France	2010	Review article	Elterman et al.	Canada	1980	Reviewed abstract only
Hawelka et al.	Germany	2010	Unsupported method	Leisman e Schwartz	USA	1978	Unsupported method
Medland et al.	UK	2010	Visual Evaluation	Adler-Grinberg e Stark	USA	1978	Visual training
Thaler et al.	Germany	2009	Population with comorbidity	Bogacz et al.	USA	1974	Age group
Kapoula et al.	France	2009	Reviewed abstract only	Zangwill e Blakemore	USA	1972	Unsupported method
Kirk	UK	2009	Visual Training				

a Articles excluded only from meta-analysis but included in the qualitative evaluation.

**Table 2:** Characteristics of included studies.

Authors	Year	Local	Apparatus/Method	N of the dyslexic group	N of the control group
Tiadi et al.^24^	2016	France	Video-oculografia (EyeBrainr T2).	55 (IM = 10.2)	55 (AA = 8.34)^a^ e 55 (AA = 10.5)^b^
Vagge et al.^25^	2015	Switzerland	Video-oculografia infravermelha (OBER 2 System)	11 (IM = 9.4)	11 (AA = 9.2)^b^
Seassau et al.^26^	2014	France	Video-oculografia	43 (IM = 10.6)	42 (AA = 10.7)^a^
Tiadi et al.^27^	2014	France	Video-oculografia infravervelha (mobileEBT®, e(ye)BRAIN)	56 (IM = 10.5)	56 (AA = 10.6)^b^
Pan et al.^28^	2013	China	Video-oculografia (EyeLink II – Eye Tracker de alta velocidade)	30 (IM = 10.7)	26 (AA = 10.6)^b^
Bucci et al.^29^	2012	USA	Video-oculografia infravervelha (mobileEBTH, e(ye)BRAIN).	12 (IM = 11)	10 (AA = 8.3)^a^ e 9 (AA = 11)^b^
Jainta e Kapoula^30^	2011	USA	Video-oculografia (Chronos Eye-tracking).	13 (IM = 11.7)	7 (AA = 12.7)^b^
Bucci et al.^31^	2007	France	Video-oculografia fotoelétrica (Oculomotor-Bouis).	18 (IM = 11.4)	13 (AA = 11.1)^b^

AA, Average age.

a Group paired for school age.

b Group paired by chronological age.

**Table 3:** Mean and standard deviation of the amplitudes of saccades for children with and without dyslexia, per study.

Authors	Average amplitude of saccades (±SD)	
	Dyslexic group	Control group
Bucci et al.^31^	27.1 ± 1	35 ± 0.8
Bucci et al.^26^	29.1 ± 1.8	36.9 ± 1.8
Jainta e Kapoula^30^	2.42 ± 0.99	2.09 ± 0.52
Tiadi et al.^24^	8.6 ± 1.3	9.7 ± 1.2

**Table 4:** Mean and standard deviation of fixation duration for children with and without dyslexia, per study.

Authors	Average length of saccades fixation (±SD) ms	
	Dyslexic group	Control group
Bucci et al.^31^	1497 ± 107	337 ± 29
Jainta e Kapoula^30^	351 ± 118.4	280.1 ± 85.1
Pan et al.^28^	322 ± 42	283 ± 30
Tiadi et al.^27^	291.2 ± 36.4	235.5 ± 34.3

**Table 5:** Mean and standard deviations of the total number of saccades for children with and without dyslexia, per study.

Authors	Average number of total saccades (±SD)	
	Dyslexic group	Control group
Bucci et al.^29^	95 ± 9	36 ± 3
Vagge et al.^25^	170.8 ± 77.7	101.9 ± 32.7

### Risk assessment of bias

The analysis of the quality of the included articles^22^, ^23^, ^24^, ^25^, ^26^, ^27^, ^28^, ^29^, ^30^, ^31^, ^32^, ^33^, ^34^ and the risk of bias associated with disease (Table 1) are shown in Table 6.

**Table 6:** Quality of articles included, according to the Newcastle-Ottawa quality assessment scale.

Authors	Representativeness of the sample	Justified sample size	Rate of non-answers	Exposure calculation	Comparability	Evaluation of results	Appropriate statistical testing	Final evaluation
Tiadi, Gérard, Peyre et al. (2016)^24^	Not representative	Yes	0	Validated tool	Yes	Own report	Yes	7/10
Lukasova, Silva and Macedo (2016)^20^	Not representative	Yes	0	Validated tool	Yes	Own report	Yes	7/10
Vagge, Cavanna, Traverso et al. (2015)^25^	Not representative	No	0	Validated tool	Yes	Own report	Yes	6/10
Bucci, Mélithe, Ajrezo et al.(2014)^21^	Not representative	Yes	0	Validated tool	Yes	Own report	Yes	7/10
Seassau, Gérard, Bui-Quoc et al. (2014)^26^	Not representative	Yes	0	Validated tool	Yes	Own report	Yes	7/10
Tiadi, Seassau, Bui-Quoc et al. (2014)^27^	Not representative	Yes	0	Validated tool	Yes	Own report	Yes	7/10
Pan, Yan, Laubrock, Hua Shu et al. (2013)^28^	Not representative	Yes	0	Validated tool	Yes	Own report	Yes	7/10
Bucci, Nassibi, Gerard et al. (2012)^29^	Not representative	No	0	Validated tool	Yes	Own report	Yes	6/10
Kraljevic and Palmovic (2011)^22^	Not representative	No	0	Validated tool	Yes	Own report	Yes	5/10
Jainta and Kapoula (2011)^30^	Not representative	No	0	Validated tool	Yes	Own report	Yes	6/10
Bucci, Gignac and Kapoula (2008)^23^	Not representative	No	0	Validated tool	Yes	Own report	Yes	6/10
Bucci, Gignac and Kapoula (2007)^31^	Not representativa	No	0	Validated tool	Yes	Own report	Yes	6/10
Santos, Behlau and Caovilla (1995)^12^	Not representative	No	0	Validated tool	Yes	Own report	Yes	6/10

Result presented in the form: points obtained/maximum score.

^a^Minimum criterion of n ≥ 30 (central limit theorem).

^b^Maximum score of 10 stars.

All studies included were characterized as observational and cross-sectional studies. In addition, at the final evaluation, all of them had a percentage of quality equal or superior to 50% (5/10) and six obtained a maximum score of 70% (7/10). The sample size of children with dyslexia was a concern for six studies^28^, ^29^, ^30^, ^31^, ^33^, ^34^ that fit the central limit theorem, with samples equal to or greater than 30 subjects. However, none of them had performed calculations to estimate the minimum size of their samples. All studies population were selected by convenience. The non-response rate was not described on the studies. All used tools for data collection were validated and comparability between the control group and the dyslexic group was also possible for all of them. The evaluation of the results was done in all the works by means of own report. Finally, all studies used appropriate statistical tests.

### Data analysis

As the studies were not randomized, the groups presented great discrepancy at the first evaluation. Thus, to avoid the phenomenon of regression to the mean, it was necessary to analyse the variations between the final and initial values of the amplitudes, total number and duration of fixation of the saccades, as well as the standard deviation associated to these variations.

### Amplitude of saccades

For the comparison of the amplitude of saccades, 4 articles (described in Table 3) were meta-analysed. The mean difference of this component for dyslexic amplitude was -3.33, with 95% CI (-6.82–0.15). The overall effect test yielded *p* < 0.06 and revealed that such a difference was not significant. The value found for the heterogeneity was I2 = 98%, with *p* < 0.00001 (Fig. 2).

### Fixation time

Four articles were submitted to metanalyses for fixation duration (described in Table 4). For this parameter, the mean interval for dyslexics was 1.19 ms with 95% CI (0.68–1.70). The overall effect test yielded *p* < 0.0001 and revealed that such difference was significant. However, a heterogeneity value I2 = 53% was found, with *p* < 0.12 (Fig. 3).

### Total number saccades

The number of articles to be meta-analysed for the total number of saccades was only 2 (described in Table 5). The mean difference of this component for the number of saccades was 59.12, with 95% CI (53.69–64.54). The overall effect test yielded *p* < 0.000001 and revealed significant difference. In addition, the heterogeneity was I2 = 0%, with *p* < 0.70 (Fig. 4), considered perfect.

## Discussion

Approximately half of the analyzed studies shows immaturity of the cortical areas that control the visual fixation system in dyslexic children when compared to children with typical development.^29^, ^32^, ^34^ In dyslexic people, the percentage with poor binocular coordination during and after the saccades, is greater than in control cases. In this way, listening to the teacher in the classroom, copying and transcribing written lessons on the blackboard, reading the books lessons, writing and concentrating become complex activities for dyslexics since they lack the integrity of oculomotor functions and vestibular interconnections.^18^, ^35^, ^36^, ^37^ A clear vision requires a quick alignment of the fovea with the object of interest (saccade) and keep it aligned to this object for a sufficient period of time so that the visual system can perform a detailed analysis of the image (fixation duration). In general, saccadic movements are interesting due to their close relation with attention. Its measurement may reveal disturbances in oculomotor activation and help diagnosis of neurological disorders.^14^

Data from the selected articles confirms the existence of a considerable difference in the ocular motor parameters between the groups. This disparate behaviour has been described in a study^31^ of 112 children with a mean age of 10.4 years, divided into two groups: 56 typical children and 56 with dyslexia. The authors compared latency values and found an increase in the dyslexic group. Similar results were found, in another French study^24^ of 30 children, with a mean age of 11.6 years, also divided into dyslexic (16) and non-dyslexic (14).

In addition, measurements of gain and mean velocities were reduced in the dyslexic groups with the occurrence of anticipatory saccades and large numbers of long-lasting, unstable fixations compared with the control group (Kraljević and Palmović 2011; Bucci et al., 2012; Bucci et al., 2004; Seassau et al., 2014). Several hypotheses were raised in order to justify such a difference: reduction or deficiency in the processing of visual attention and the precision of the search; immaturity of the saccadic ocular interaction and vergence systems; and the possibility of visual/ocular motor imperfections were frequently cited.

The impairment in convergence, ocular saccadic interaction, and fusion capacity of divergence may be due to a lack of cortical maturation, and the poor oculomotor performance in children with dyslexia is possibly related to a deficit in the allocation of visual attention, suggesting a processing deficiency.^22^, ^23^, ^24^, ^28^, ^33^

Learning disability, related to dyslexia, can still be justified by the interaction between the saccadic and vergence subsystems, on which knowledge acquisition may be based.^27^

VNG is a computerized system that uses the principle of capturing eye movements by infrared sensors placed on special glasses or mask. The movements are measured and analyzed on a video monitor and further recorded. In VENG, the most commonly used method for recording ocular movements, the variation of corneal-retinal potential, is used to record and analyze features of the vestibulo-ocular reflex and saccadic visual systems involved in the persecution, optokinesis and fixation.^18^, ^36^

The use of VNG and VENG exams has already been consolidated in several pathologies that involve the cerebral cortex, the brainstem, the superior colliculus, the basal ganglia, the cerebellum and the sensory organs (semicircular canals and otolithic organs). This neural system controls the ocular fixation in various conditions such as the movement of the visual object, movement of the observer and changes in vision. Among the most common pathologies associated with this network are: petrositis (infection in the petrous part of the temporal bone that can reach the fourth cranial nerves, multiple sclerosis (demyelinating immune disease), optic neuritis (visual or subacute visual loss (vogt-koyanagi-harada syndrome, an autoimmune disease affecting eyes, skin, ears, and meninges) and congenital rubella (viral infection by vertical transmission, which mainly affects vision, hearing and the cardiac function of a newborn). Scientific and clinical evidence should be reasons to extend the use of these exams to the differential diagnosis on learning disorders, such as dyslexia, which does not yet have specific neurobiological cause described in the literature.^13^, ^38^, ^39^, ^40^, ^41^

When speaking of otoneurological evaluation we usually refer to practices performed by the speech-language pathologist related to the investigation of auditory and vestibular function, with the objective of assisting in the diagnosis of the disturbances of balance. However, the range of possibilities offered by the instruments such as VENG to the researcher is immense, especially from the perspective of making the diagnosis of language disorders more accurate, as in the case of dyslexia.^42^

It turns out, however, that the qualitative analysis of the studies of this review have not showed data or discussion about vestibular complaints in dyslexic population. This must be due to the lack of research using VNG and VENG for these purposes. These tests are fundamentally recommended to people with vestibular problems, and it is important to reinforce the need for more studies.

## Conclusion

The study revealed that children with dyslexia have longer duration of fixation and fewer saccades during ocular movements on vectoelectro-nystagmography and video-nystagmography when compared to children without dyslexia. However, all these findings are not intended to reduce dyslexia to a purely ocular disorder, since, as noted above, it is a complex disorder that may be associated with deficits in executive functions, difficulties in auditory and/or visual processing and psychomotor development.^1^

## Conflicts of interest

The authors declare no conflicts of interest.
