# Supplementary material for: Ocular findings from otoneurological examinations in children with and without dyslexia: a systematic review with meta-analysis
Source: Braz J Otorhinolaryngol. 2021 Nov 25;88(Suppl 3):S192–201. doi: 10.1016/j.bjorl.2021.10.006 (PMC9760992; doi:10.1016/j.bjorl.2021.10.006)
Supplement: Supplementary file 1 [file mmc1.doc]

**BJORL-D-21-00256_Supplementary Material**

**Table 1 Supplementary** Literature research strategy used for all databases.

| Medline (via PubMed) #1 E #2 |
| --- |
| #1 (dyslexia) OR (written reading disorder) OR (developmental reading disorder) OR (word blindness) OR (developmental dyslexia) OR (reading disorder) OR (alexia) OR (language disorder) |
| #2 (otoneurology) OR (videonystagmography) OR (vectoelectronystagmography) OR (ocular tests) OR (ocular evidence) OR (nystagmus) OR (eye movement) OR (nystagmus semi spontaneous) OR (optokinetic nystagmus) OR (pendular tracking) OR (saccades) |
| SciELO/WorldCat /Lilacs/CENTRAL/OpenGrey.eu and other data sources (dyslexia OR written reading disorder OR developmental reading disorder OR word blindness OR developmental dyslexia OR reading disorder OR alexia OR language disorder) AND (otoneurology OR videonystagmography OR vectoelectronystagmography OR ocular tests OR ocular evidence OR nystagmus OR eye movement OR nystagmus semi spontaneous OR optokinetic nystagmus OR pendular tracking OR saccades) |

**Table 2 Supplementary** Newcastle-Ottawa Scale (adapted) for quality evaluation of cross-sectional studies.

| **Rating:** (Maximum 5 stars) |
| --- |
| 1. Representativeness of the sample: |
| a) Truly representative of the mean in the target population. *(All subjects or random sampling). |
| b) A little representative of the average in the target population. *(Non-random sampling). |
| c) Group of users selected. |
| d) Description of the sampling strategy. |
| 2. Sample Size: |
| a) Justified and satisfactory.* |
| b) Not justified. |
| 3. No answers: |
| (a) Comparability between responses and non-responses is established and the response rate is satisfactory.* |
| (b) the response rate is not satisfactory or the comparability between the responses and the non-responders is unsatisfactory. |
| c) Description of the response rate or characteristics of responses and non-responses. |
| 4. Exposure calculation (risk factor): |
| a) Validated measurement tool.** |
| b) Not validated measuring tool, but the tool is available or described.* |
| c) Description of the measuring tool. |
| **Comparability:** (Maximum of 2 stars) |
| 1. Objects in different result groups are comparable, based on study design or analysis. Confounding factors are controlled. |
| a) The study takes into account the most important factor (select one).* |
| b) Control of the study for any additional factors.* |
| **Result:** (Maximum 3 stars) |
| 1. Evaluation of results: |
| a) Independent blind rating.** |
| b) Relationship of records.** |
| c) Own report.* |
| d) No description. |
| 2. Statistical test: |
| a) The statistical test used to analyze the data are clearly described and adequate and the measurement of the association is presented, including confidence intervals and the probability level (p-value).* |
| b) The statistical test is not appropriate, not described or incomplete. |
| This scale was adapted from the Newcastle-Ottawa Quality Score Scale for cohort studies to make an evaluation of cross-sectional quality studies for the systematic review “Are workers' health concerns to vaccinate related to their knowledge, beliefs and attitudes? A systematic review”. |
